# Genetic and Clinical Characteristics of Chinese Adult Patients With Krabbe Disease

**DOI:** 10.1002/cns.70708

**Published:** 2025-12-18

**Authors:** Yi Zhang, Hui‐Fen Huang, Juan‐Juan Xie, Wang Ni, Hao Yu, Zhi‐Ying Wu

**Affiliations:** ^1^ Department of Medical Genetics and Center for Rare Diseases, Second Affiliated Hospital Zhejiang University School of Medicine, and Zhejiang Key Laboratory of Rare Diseases for Precision Medicine and Clinical Translation Hangzhou China; ^2^ Nanhu Brain‐Computer Interface Institute Hangzhou China; ^3^ Department of Neurology, Lishui Hospital Zhejiang University School of Medicine Lishui China; ^4^ MOE Frontier Science Center for Brain Science and Brain‐Machine Integration, School of Brain Science and Brain Medicine Zhejiang University Hangzhou China

**Keywords:** clinical features, GALC, genetic spectrum, Krabbe disease

## Abstract

**Aim:**

This study aims to expand the clinical and genetic spectrum of Krabbe disease (KD) in Chinese adult patients and to improve diagnosis and understanding of its phenotypic diversity.

**Methods:**

Patients clinically suspected of leukodystrophy were recruited between 2015 and 2025. Clinical features were collected, and whole‐exome sequencing (WES) was performed to identify potential variants. The pathogenicity of detected variants was classified according to the American College of Medical Genetics and Genomics (ACMG) standards and guidelines. Functional assays assessing protein expression, processing, secretion, subcellular localization, and enzymatic activity were conducted to further validate variant pathogenicity.

**Results:**

Fourteen unrelated patients were genetically diagnosed with KD, and their genetic and clinical features were summarized. Eleven variants in *GALC* were identified, including a novel missense variant c.1019C>T (p.P340L) which is not reported in the Human Gene Mutation Database (HGMD). Unlike most adult patients who typically present with spastic paraplegia, the patient carrying this variant exhibited initial symptoms of peripheral neuropathy. Functional experiments demonstrated that the variant led to impaired protein processing and localization, as well as reduced GALC enzymatic activity. Other variants including p.D56H, p.L377X, p.L441X, and p.L634S also affected GALC functions to varying degrees.

**Conclusion:**

This study enhances the genotypic and phenotypic characterization of KD in China, aiding in differential diagnosis and genetic counseling. Functional data reinforce the pathogenicity of identified variants.

## Graphical abstract

This study presents a comprehensive analysis of the genetic and clinical spectrum of Krabbe disease in a Chinese cohort, with a particular focus on adult‐onset cases. We identified 11 *GALC* variants, including the novel mutation p.P340L, and provided functional evidence supporting its pathogenicity through impaired protein processing, mislocalization, and reduced enzymatic activity. The clinical heterogeneity observed highlights the diagnostic challenge of adult‐onset Krabbe disease.

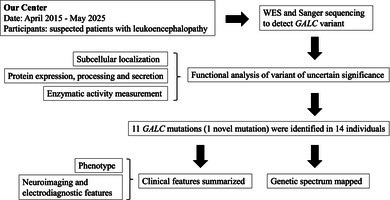

## 1 Introduction

**FIGURE 1:**
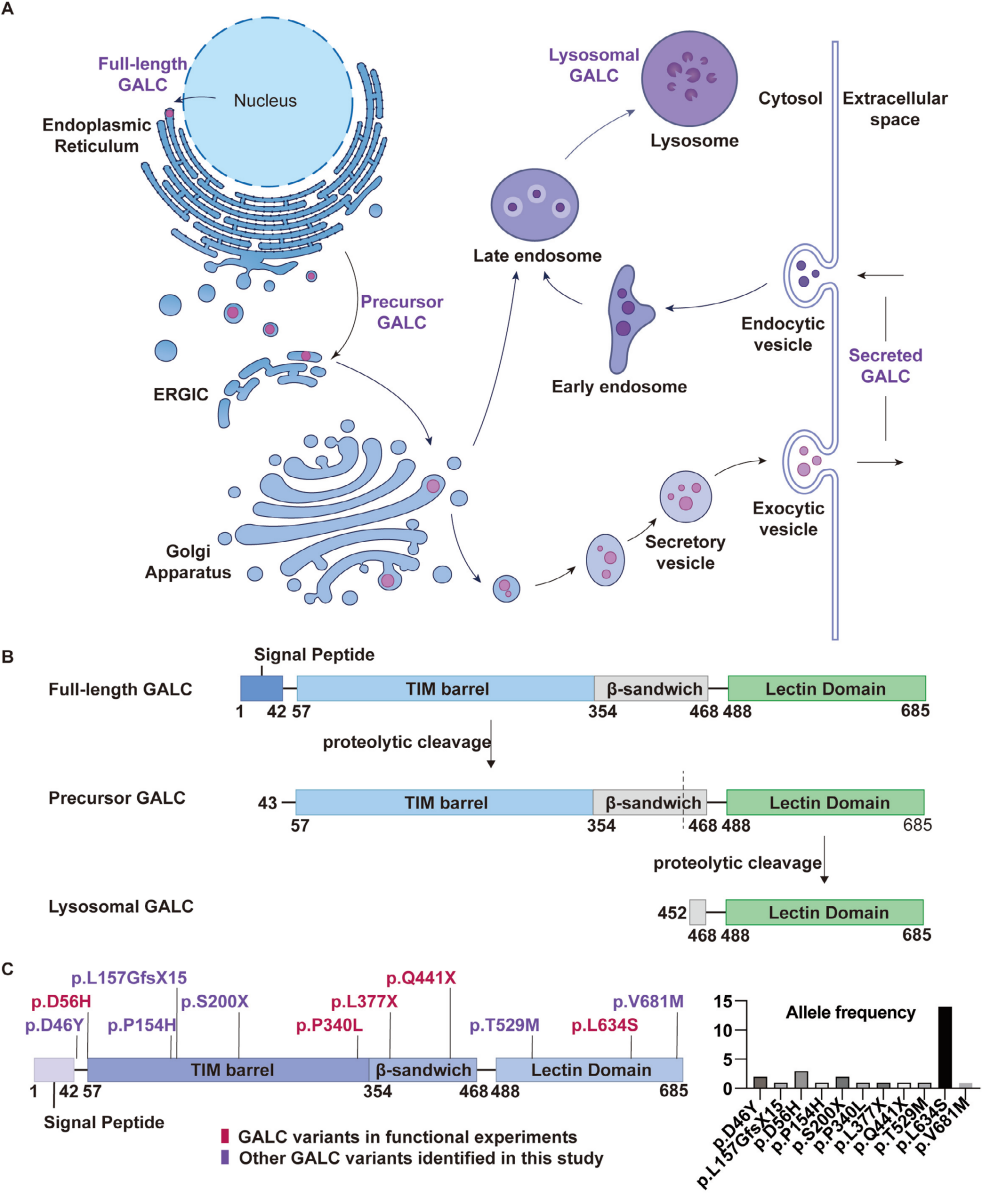
Schematic diagram of the *GALC* gene. (A, B) Synthesis, processing, and trafficking of the GALC protein. The full‐length GALC protein undergoes proteolytic cleavage in the endoplasmic reticulum to remove the signal peptide and is post‐translationally modified in the Golgi apparatus to form precursor GALC, which is secreted or transported to lysosomes. Key structural domains are indicated: signal peptide (SP) (1–42 aa), TIM barrel domain (57–353 aa), β‐sandwich domain (354–468 aa), and lectin‐binding domain (488–685 aa). (C) Distribution and allele frequency of variants identified in this cohort. Variants subjected to functional experiments are labeled in red; others are in purple. The p.L634S variant is a hotspot in this cohort. ERGIC, endoplasmic reticulum‐Golgi intermediate compartment; TIM, triosephosphate isomerase.

Krabbe disease (KD) (OMIM 245200), also known as globoid cell leukodystrophy (GLD), is an autosomal recessive lysosomal storage disorder caused by mutations in the *GALC* gene, which encodes a lysosomal galactocerebrosidase. The GALC protein contains three main domains: the triosephosphate isomerase (TIM) barrel domain, the β‐sandwich domain, and the lectin‐binding domain. This glycoprotein is processed in the endoplasmic reticulum (ER) and the Golgi apparatus to form precursor GALC (pre‐GALC), which is subsequently secreted via secretory vesicles or transported to lysosomes through the endosomal pathway. Within lysosomes, GALC undergoes proteolytic cleavage to generate an N‐terminal fragment (~50 kDa) and a C‐terminal fragment (~30 kDa) (Figure 1A,B) [1]. The C‐terminal fragment represents the mature form of GALC responsible for degrading galactolipids such as galactosylceramide and psychosine in lysosomes [2, 3]. These galactolipids play a vital role in myelin production. Mutations in *GALC* disrupt normal protein processing, secretion, and localization, ultimately impairing galactolipid metabolism. The accumulation of these substrates leads to the formation of globoid cells and disrupts myelination, resulting in demyelination of both the central and peripheral nervous systems [4].

KD has a low worldwide incidence, estimated at approximately 1 in 100,000 individuals [5]. The disease exhibits highly variable severity and progression, with clinical onset ranging from the first few weeks of life to late infantile, juvenile, and adult stages. KD is commonly classified into five forms: early‐infantile (onset 0–6 months), late‐infantile (7–12 months), later‐onset (13 months to 10 years), adolescent (11–20 years), and adult (from 21 years onward) [6]. The infantile form, which is the most common, typically presents within the first 6 months of life with symptoms such as developmental delay, limb spasticity, axial hypotonia, seizures, visual impairment, and irritability. Disease progression is rapid, and most affected children succumb to respiratory failure or complications by 2 years of age [7]. The adult form, with later onset, may manifest during adolescence or adulthood. Adult‐onset KD accounts for less than 10% of all cases and is often misdiagnosed due to its heterogeneous presentation. Symptoms are more varied and may include motor impairment such as lower limb spasticity and gait abnormality, loss of manual dexterity and reduced fine motor skills, burning paresthesia and peripheral neuropathy, psychiatric symptoms and cognitive impairment [8]. Disease progression is slower in adults but can still lead to significant disability and death.

The diagnosis of KD is confirmed through molecular genetic analysis of the *GALC* gene. Measurement of GALC activity in leukocytes isolated from whole blood or cultured skin fibroblasts can support diagnosis, although it does not indicate clinical type or prognosis [7, 9]. Additional auxiliary examinations, such as brain magnetic resonance imaging (MRI) and electromyography (EMG), also reveal characteristic abnormalities. While infantile‐onset KD often shows distinct MRI abnormalities in the deep cerebral white matter, dentate nucleus, and cerebellar white matter, juvenile‐ and adult‐onset forms typically exhibit cerebral atrophy and T2 hyperintensities in the parieto‐occipital regions or corticospinal tracts [6, 7, 10]. EMG findings commonly include prolonged conduction latency, decreased amplitude, and slowed conduction velocity in motor and sensory nerves [11].

The highly variable clinical presentation of adult‐onset KD often leads to misdiagnosis and underestimation of its incidence. Additionally, the mutation spectrum of *GALC* in adult‐onset cases differs from that in infantile forms and varies across populations. To further expand the clinical and genetic spectrum of KD in China, we compiled and summarized cases from our center. This study reports 11 variants in *GALC* identified in 14 probands, including four previously described cases [12]. Functional experiments were conducted to confirm the pathogenicity of the variants identified in this and previous studies.

## 2 Materials and Methods

### 2.1 Subjects

Patients clinically diagnosed with white matter lesions were collected between April 2015 and May 2025. After targeted sequencing or whole exome sequencing, patients carrying pathogenic variants of causative genes from other diseases with white matter involvement, including CADASIL and several types of leukodystrophy, were excluded. Fourteen pedigrees were recruited in this study since they carried compound heterogeneous or homogeneous variants, among which four pedigrees were reported in our previous work. Clinical and genetic data were summarized and analyzed.

### 2.2 Genetic Analysis

Genomic DNA was extracted from peripheral EDTA‐treated blood using the QlAamp DNA Blood Minikit (QlAGEN, Hilden, Germany). Whole exome sequencing (WES) was performed on the lllumina HiSeq X Ten platform (XY Biotechnology Co. Ltd.) using Agilent SureSelectTM Human All Exon v6 kit. Detected variants were further classified according to the American College of Medical Genetics and Genomics (ACMG) standards and guidelines [13]. Population frequencies of the variants were obtained from the 1000 Genomes Project (1000 g), Exome Aggregation Consortium (ExAC), and the Genome Aggregation Database (gnomAD). Pathogenicity prediction was performed by Sorting Intolerant From Tolerant (SIFT), Polymorphism Phenotyping v2 (Polyphen‐2), Mutation Taster, Rare Exome Variant Ensemble Learner (REVEL), and MetaLR. Suspected variants were further confirmed by Sanger sequencing as described in our previous works.

### 2.3 Plasmid Construction

The full‐length coding sequence of the human wild‐type *GALC* gene (NM_000153) was cloned into the p3 × flag‐CMV vector, with an HA tag linked to the C‐terminus. Variants were introduced into the above plasmids by PCR mutagenesis with 2 × Phanta Max Master Mix (P515, Vazyme). All plasmids were fully sequenced after construction or mutagenesis.

### 2.4 Cell Culture and Transient Transfection

HEK293T cells and U2OS cells were cultured at 37°C under 5% CO_2_ in DMEM (C11995500CP, Gibco) supplemented with 10% fetal bovine serum (30044333, Gibco). Cells were seeded in 12‐well plates and transfected with 1 μg of *GALC* plasmid mediated by Lipofectamine 3000 (L3000015, Invitrogen). The pEGFP‐C1 plasmid was co‐transfected to ensure consistent transfection levels. For protein expression analysis, the media was replaced with serum‐free conditioned media 6 h post‐transfection. Culture media and cells were collected 48 h post‐transfection. For immunofluorescence analysis, cells were harvested 24 h post‐transfection.

### 2.5 Protein Extraction and Western Blot

Secreted proteins were extracted by methanol‐chloroform precipitation as previously described [14]. Total protein was extracted from HEK293T cells 48 h post‐transfection by RIPA lysis buffer (P0013B, Beyotime) with 1 mM PMSF. Protein samples were separated by 10% SDS‐PAGE and then transferred to PVDF membranes. Antibodies used were anti‐Flag (M1403‐2, Huabio, 1:5000), anti‐GALC‐N (A3873, Abclonal, 1:1000), anti‐GALC‐C (11991‐1‐AP, Proteintech, 1:1000), anti‐GAPDH (AC035, Abclonal, 1:1000), and anti‐GFP (sc9996, Santa Cruz, 1:400). Bands were analyzed using Image J software.

### 2.6 Immunofluorescence

U2OS cells were seeded on glass coverslips in 12‐well plates and transfected with Flag‐tagged *GALC* plasmids. After 24 h, cells were fixed with 4% paraformaldehyde for 15 min at room temperature, permeabilized with PBS containing 0.1% Triton X‐100 for 10 min and then blocked in PBS containing 0.1% Triton X‐100 and 10% goat serum albumin (16210064, Gibco) for 60 min. Cells were incubated with anti‐HA (05‐904, Millipore, 1:1000), anti‐GALC‐C (11991‐1‐AP, Proteintech, 1:1000), anti‐KDEL (ab176333, Abcam, 1:200), anti‐TGN46 (ab50595, Abcam, 1:200) and anti‐Lamp1 (9091s, Cell Signaling Technology, 1:200; ab25630, Abcam, 1:50) in blocking buffer at 4°C overnight, followed by secondary anti‐mouse or anti‐rabbit IgG Alexa Fluor 488/594 (1:1000, Life Technologies) for 2 h at room temperature. Nuclei were stained with Hoechst (Coolaber, SL7130). Fluorescence images were captured using a Zeiss LSM900 system. To analyze co‐localization, intensities were measured and Pearson's correlation coefficients were calculated using Fiji software.

### 2.7 Enzymatic Activity Assay

Lysosomal Galactocerebrosidase (GALC) Analysis Kit (ab253371, Abcam) was used to measure the GALC activity according to the manufacturer's protocol. Fluorescence was measured on the Thermo Varioskan Flash microplate reader at excitation/emission wavelength of 365/454 nm.

### 2.8 Statistical Analysis

Statistical analyses were performed and graphed using SPSS version 26.0 and GraphPad Prism 10. Experimental data were presented as the mean ± standard deviation (SD). The data normality was assessed by the Shapiro–wilk test and one‐way ANOVA and Dunnett's multiple comparison were used for comparison. Values of *p* < 0.05 were considered statistically significant.

## 3 Results

### 3.1 Genetic Analysis of Variants in *GALC*

**FIGURE 2:**
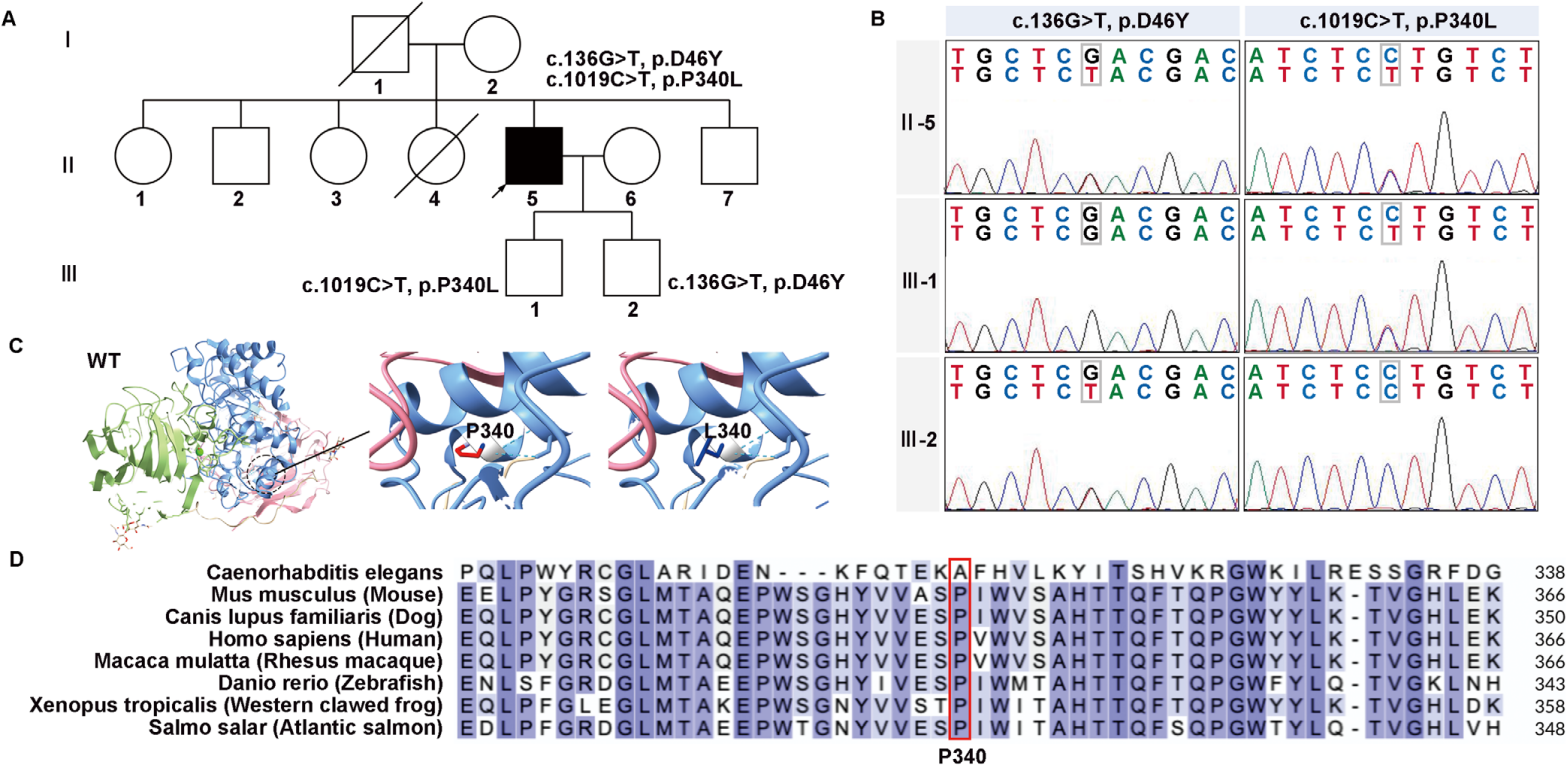
Genetic analysis of the novel *GALC* missense variant p.P340L identified in proband 6. (A) Pedigree of the family carrying p.P340L. Open symbols: unaffected; filled symbols: affected; squares: male; circles: female; arrow: proband. (B) Sanger sequencing chromatograms of the proband and his sons, confirming co‐segregation. (C) Homology modeling of GALC. Wild‐type GALC (based on PDB 3ZR5) and the P340 residue in the TIM barrel domain are shown. (D) Multiple sequence alignment of GALC orthologues. Evolutionary conservation of Pro340 is shown. WT, wild‐type.

Whole‐exome sequencing (WES) followed by Sanger validation identified a total of 11 variants in *GALC* among 14 probands (Table 1). The distribution of these variants and their allele frequencies within the cohort were illustrated in Figure 1C. Among these, c.1019C>T (p.P340L) was a novel variant that is not reported in HGMD. It was detected in proband 6 and confirmed by Sanger sequencing (Figure 2A) and co‐segregation analysis within the family (Figure 2B). The variant was absent from 1000 g and had a frequency of 8.29 × 10^−6^ in ExAC and 8.03 × 10^‐6^ in gnomAD. *In silico* predictions using PolyPhen‐2, MutationTaster, REVEL, and MetaLR consistently indicated damaging effects on protein function. Previous studies have shown that the TIM barrel domain contains the main binding surface of GALC [15]. Homology modeling revealed that p.P340L was located within the TIM barrel near the substrate‐binding interface, suggesting potential effects on protein stability or activity (Figure 2C). Multiple sequence alignment indicated high evolutionary conservation of the Pro340 residue (Figure 2D). The variants c.166G>C (p.D56H) and c.1321C>T (p.Q441X) were first described in our previous work [12]. According to ACMG classification, c.136G>T (p.D46Y), c.467_468dup (p.L157GfsX15), c.599C>A (p.S200X), c.1130Tdel (p.L377X), c.1321C>T (p.Q441X), c.1586C>T (p.T529M), c.1901T>C (p.L634S) were classified as pathogenic variants and c.461C>A (p.P154H), c.2041G>A (p.V681M) were classified as likely pathogenic variants. c.166G>C (p.D56H), and c.1019C>T (p.P340L) were classified as variant of uncertain significance (VUS).

**TABLE 1:** Eleven variants identified in 14 probands with Krabbe disease.

Exon	Nucleotide change	Amino acid change	Domain	In silico prediction					Frequency of population (all; East Asian)			ACMG
				SIFT	Polyphen‐2	Mutation Taster	REVEL	MetaLR	1000 g	ExAC	gnomAD	
1	c.136G>T	p.D46Y	—	D	PD	D	D	D	—; —	0.0003; 0.0025	5.91E‐05; 0.0006	P (PS3 + PM2 + PM3 + PP3 + PP4 + PP5)
1	c.166G>C	p.D56H	—	D	PD	D	D	D	—; —	—; —	—; —	VUS (PM2 + PP3 + PP4) → LP (PS3 + PM2 + PP3 + PP4)
5	c.461C>A	p.P154H	TIM barrel	D	PD	D	D	D	—; —	8.31E‐06; 0.0001	1.20E‐05; 0.0002	LP (PM2 + PM3 + PP3 + PP4 + PP5)
5	c.467_468dup	p.L157GfsX15	TIM barrel	—	—	—	—	—	—; —	—; —	—; —	P (PVS1 + PM2 + PM4)
6	c.599C>A	p.S200X	TIM barrel	—	—	—	—	—	—; —	—; —	—; —	P (PVS1 + PM2 + PM3 + PP3 + PP4 + PP5)
9	**c.1019C>T**	**p.P340L**	TIM barrel	T	PD	D	D	D	—; —	8.29E‐06; 0.0001	8.03E‐06; 0.0001	VUS (PM2 + PM3 + PP3) → P (PS3 + PM2 + PM3 + PP3)
10	c.1130Tdel	p.L377X	β‐sandwich	—	—	—	—	—	—; —	—; —	—; —	P (PVS1 + PM2 + PM3 + PP3 + PP4 + PP5)
12	c.1321C>T	p.Q441X	β‐sandwich	—	—	D	—	—	—; —	—; —	—; —	P (PVS1 + PM2 + PM3 + PP3 + PP4 + PP5)
14	c.1586C>T	p.T529M	Lectin domain	T	PD	D	D	D	—; —	6.64E‐05; 0	0.0001; 0	P (PS3 + PM2 + PM3 + PP3 + PP4 + PP5)
16	c.1901T>C	p.L634S	Lectin domain	D	PD	D	D	D	0.00159744; 0.0079	0.0007; 0.0090	0.0007; 0.0085	P (PS3 + PM2 + PM3 + PP3 + PP4 + PP5)
17	c.2041G>A	p.V681M	Lectin domain	D	PD	D	D	D	0.000199681; —	0.0002; 0.0008	0.0002; 0.0005	LP (PM2 + PM3 + PP3 + PP4 + PP5)

*Note:* Novel variant is in bold. →, re‐classification of variant after functional studies.

Abbreviations: 1000 g, 1000 Genomes Project; ACMG, American College of Medical Genetics and Genomics; D, damaging or disease‐causing; ExAC, Exome Aggregation Consortium; gnomAD, The Genome Aggregation Database; LP, likely pathogenic; P, pathogenic; PD, probably damaging; Polyphen‐2, Polymorphism Phenotyping v2; REVEL, Rare Exome Variant Ensemble Learner; SIFT, Sorting Tolerant From Intolerant; T, tolerated; TIM, triosephosphate isomerase.

### 3.2 Protein Processing and Secretion of Variants in *GALC* Were Impaired

**FIGURE 3:**
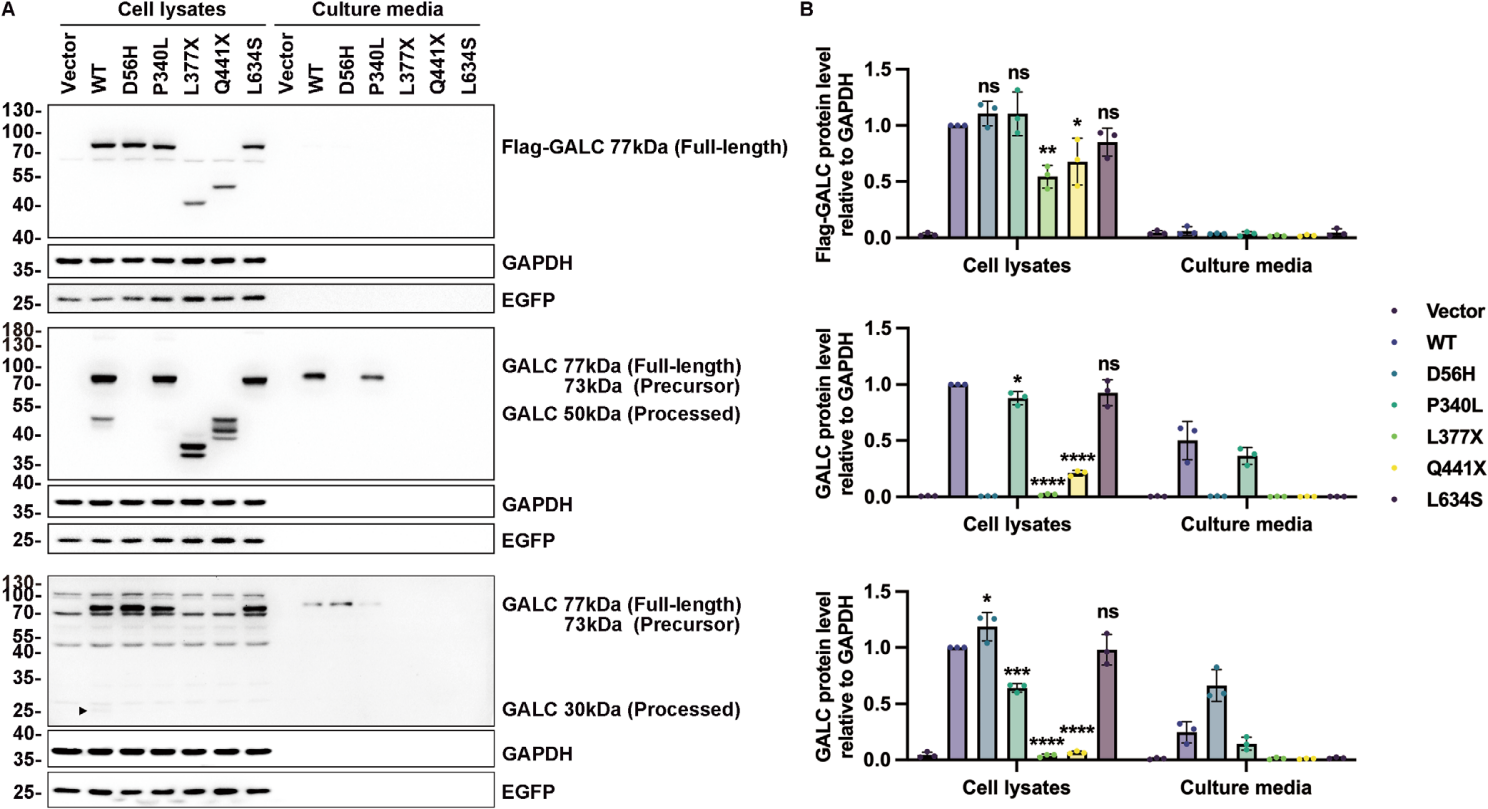
Protein expression analysis of GALC variants. (A) Immunoblot of Flag‐tagged GALC variants in whole‐cell lysates and conditioned media from HEK293T cells. Blots were probed with anti‐Flag (top), anti‐GALC‐N (middle), and anti‐GALC‐C (bottom) antibodies. Processed fragments were detected only in WT; the 30 kDa fragment is indicated by a black triangle. EGFP served as a transfection control; GAPDH was a loading control. (B) Quantification of relative expression levels of Flag‐tagged GALC in total protein, normalized to GAPDH and compared to WT. *p* values: 0.1234 (ns), 0.0332 (*), 0.0021 (**), 0.0002 (***), < 0.0001 (****). Error bars represent mean ± SD (*n* = 3 independent experiments). WT, wild‐type.

According to ACMG guidelines, functional studies supportive of a damaging effect on the gene or gene product provide important evidence of the variant classification. To further validate the pathogenicity of variants in *GALC*, particularly the VUS ones, functional experiments were conducted. Variants first identified by our center (D56H, P340L, Q441X) and one reported by another Chinese group (L377X) were included [16]. The L634S variant, common in Asian populations, was used as a positive control. HEK293T cells were transfected with Flag‐tagged *GALC* plasmids to assess protein expression levels (Figure 3A). Firstly, full‐length GALC proteins (77 kDa) were detected using anti‐Flag antibodies. Compared to wild‐type (WT) GALC, the protein levels of the stop‐gain variants L377X and Q441X were markedly reduced, consistent with nonsense‐mediated decay (Figure 3B). The full‐length precursor protein levels of D56H, P340L, and L634S were comparable to WT, indicating that these variants did not affect protein synthesis or translation initiation. Next, we used two anti‐GALC antibodies targeting the N‐terminus (anti‐GALC‐N) and C‐terminus (anti‐GALC‐C) respectively to assess the GALC processing. Interestingly, the variant D56H was not detected by anti‐GALC‐N, possibly due to epitope masking. However, when immunoblotted with anti‐GALC‐C, the D56H variant exhibited a slight yet consistent increase in protein abundance. We hypothesize that this variant at the signal peptide cleavage site may mildly impair processing efficiency, which could inadvertently enhance the stability of the precursor or mature protein, leading to its accumulation. Conversely, the P340L variant showed a reduction compared to WT protein. This is likely attributable to protein misfolding caused by the variant within the substrate‐binding domain, resulting in recognition by the ER quality control machinery and subsequent degradation. The L377X and Q441X variants were detectable only with anti‐GALC‐N and showed significantly decreased levels, with additional bands indicating protein degradation. The L634S variant demonstrated mature protein levels equivalent to WT, confirming that its synthesis and initial maturation proceed normally. However, the N‐terminal 50 kDa and the C‐terminal 30 kDa fragments were only detected in WT GALC, indicating that all the variants altered the normal cleavage of GALC.

Proteins in conditioned culture media were also extracted simultaneously to verify whether secretion was affected by the variants. Analysis of secreted proteins showed that D56H was secreted at higher levels than WT, consistent with its increased stability and potential misrouting. P340L was detected at lower levels, representing a small fraction that evades ERAD and is passively secreted. Despite robust intracellular expression, the L634S variant was undetectable in the concentrated media. We propose that the loss of the lysosomal targeting signal diverts this variant entirely into the constitutive secretory pathway.

### 3.3 Subcellular Location of GALC Proteins Were Altered

**FIGURE 4:**
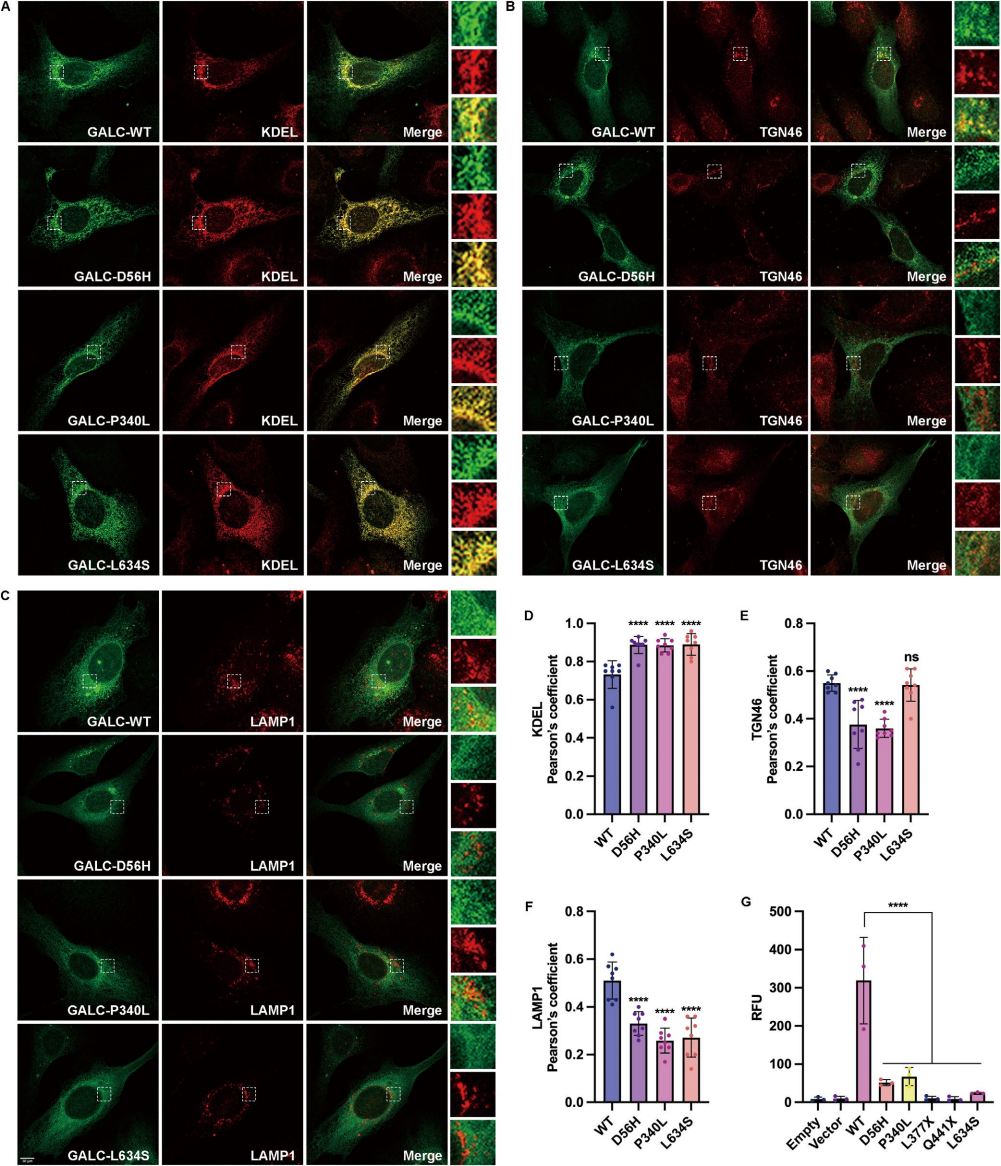
Subcellular location analysis and enzymatic activity of GALC variants. (A–C) Confocal microscopy images of U2OS cells transfected with Flag‐tagged WT and mutant GALC variants. Cells were immunostained with anti‐HA and ER marker KDEL (A), Golgi marker TGN46 (B), or anti‐GALC and lysosomal marker Lamp1 (C). Images were captured with a 63× oil immersion objective. Scale bar: 10 μm. (D–F) Pearson's correlation coefficients for co‐localization of variants with organelle markers. (G) Enzymatic activity of GALC variants measured in transfected HEK293T cells using a fluorogenic substrate. All variants showed significantly reduced activity. *p* values as in Figure 3. Error bars represent mean ± SD (*n* = 3 independent experiments). RFU, relative fluorescence units.

Subcellular localization studies provided further mechanistic insights into the trafficking deficits imposed by missense variants (D56H, P340L, and L634S). The precursor form of GALC was detected using an anti‐HA antibody, and lysosomal GALC was detected with anti‐GALC‐C. For subcellular organelle markers, KDEL was used as the ER marker, TGN46 as the Golgi apparatus marker, and Lamp1 as the lysosomal marker. Quantitative co‐localization analysis demonstrated significantly increased Pearson's coefficients for all three variants with an ER marker, indicating substantial ER retention. For the D56H variant, this is consistent with impaired signal peptide cleavage delaying its ER exit. The P340L variant exhibited ER retention, a hallmark of protein misfolding and engagement by the ER quality control apparatus. The L634S variant also displayed ER retention, which may suggest minor folding inefficiencies or retrotranslocation delays (Figure 4A,D). While the D56H and P340L variants showed reduced co‐localization with the Golgi apparatus, the L634S variant localized to the Golgi as efficiently as the wild‐type, confirming that its ER export and Golgi trafficking are uncompromised (Figure 4B,E). The most critical finding was the complete absence of co‐localization for any of the variants with lysosomal markers. This collective failure to reach the lysosome, the site of its physiological action, underscores the pathological consequence of all three variants (Figure 4C,F). The divergent trafficking fates highlight distinct molecular mechanisms converging on a common loss of function.

### 3.4 Enzymatic Activities of GALC Variants Were Significantly Reduced

The functional integrity of mutant GALC proteins was assessed by measuring enzymatic activity using a fluorogenic substrate assay in transfected HEK293T cells. Fluorescence intensity was quantified at excitation/emission wavelengths of 365 nm/454 nm. All variants showed a significant reduction in GALC enzymatic activity relative to WT (Figure 4G). These results confirmed that all variants impair GALC enzymatic activity, supporting their pathogenicity. The two VUS variants c.166G>C (p.D56H) and c.1019C>T (p.P340L) can now be determined to be likely pathogenic and pathogenic after re‐evaluation, respectively.

### 3.5 Clinical Characteristics of KD Probands

The 14 unrelated KD probands (9 male and 5 female) were systematically characterized, comprising one early‐onset case and thirteen adolescent/adult‐onset cases. A comprehensive summary of individual clinical profiles, including age of onset and major symptoms, is provided in Table 2.

**TABLE 2:** Clinical features of 14 patients with Krabbe disease.

Patient No.	Gender	AAD	AAO	Phenotype	Genetic findings	Genotype	Initial symptom	Other symptoms
1^a^	Male	28	26	Adult	c.1901T>C, p.L634S c.2041G>A, p.V681M	Compound heterozygous	Gait disturbance and difficulty walking	/
2^a^	Male	21	1	Late‐infantile	c.599C>A, p.S200X c.1586C>T, p.T529M	Compound heterozygous	Epileptic seizures	Gait disturbance and difficulty walking, developmental delay
3^a^	Female	47	35	Adult	c.166G>C, p.D56H c.461C>A, p.P154H	Compound heterozygous	Gait disturbance and difficulty walking	Lower limb weakness
4	Male	25	16	Adolescent	c.1130Tdel, p.L377X c.1901T>C, p.L634S	Compound heterozygous	Gait disturbance and difficulty walking	/
5^a^	Male	28	11	Adolescent	c.1321C>T, p.Q441X c.1901T>C, p.L634S	Compound heterozygous	Gait disturbance and difficulty walking	Dysarthria
6	Male	56	46	Adult	c.136G>T, p.D46Y **c.1019C>T, p.P340L**	Compound heterozygous	Upper limb weakness	Upper limb paresthesia
7	Male	48	48	Adult	c.1901T>C, p.L634S c.1901T>C, p.L634S	Homozygous	Gait disturbance and difficulty walking	Dysphagia
8	Male	68	28	Adult	c.461C>A, p.P154H c.1901T>C, p.L634S	Compound heterozygous	Gait disturbance and difficulty walking	Lower limb weakness
9	Female	28	16	Adolescent	c.1901T>C, p.L634S c.1901T>C, p.L634S	Homozygous	Gait disturbance and difficulty walking	/
10	Male	34	31	Adult	c.136G>T, p.D46Y c.1901T>C, p.L634S	Compound heterozygous	Gait disturbance and difficulty walking	/
11	Female	58	48	Adult	c.599C>A, p.S200X c.1901T>C, p.L634S	Compound heterozygous	Gait disturbance and difficulty walking	/
12	Male	24	19	Adolescent	c.461C>A, p.P154H c.1901T>C, p.L634S	Compound heterozygous	Gait disturbance and difficulty walking	Lower limb weakness, loss of manual dexterity
13	Female	35	35	Adult	c.1901T>C, p.L634S c.1901T>C, p.L634S	Homozygous	Lower limb weakness and numbness	/
14	Female	40	30	Adult	c.467_468dup, p.L157GfsX15 c.1901T>C, p.L634S	Compound heterozygous	Gait disturbance and difficulty walking	/

*Note:* Novel variant is in bold.

Abbreviations: AAD, age at diagnosis; AAO, age at onset.

^a^ Previously reported patients.

**FIGURE 5:**
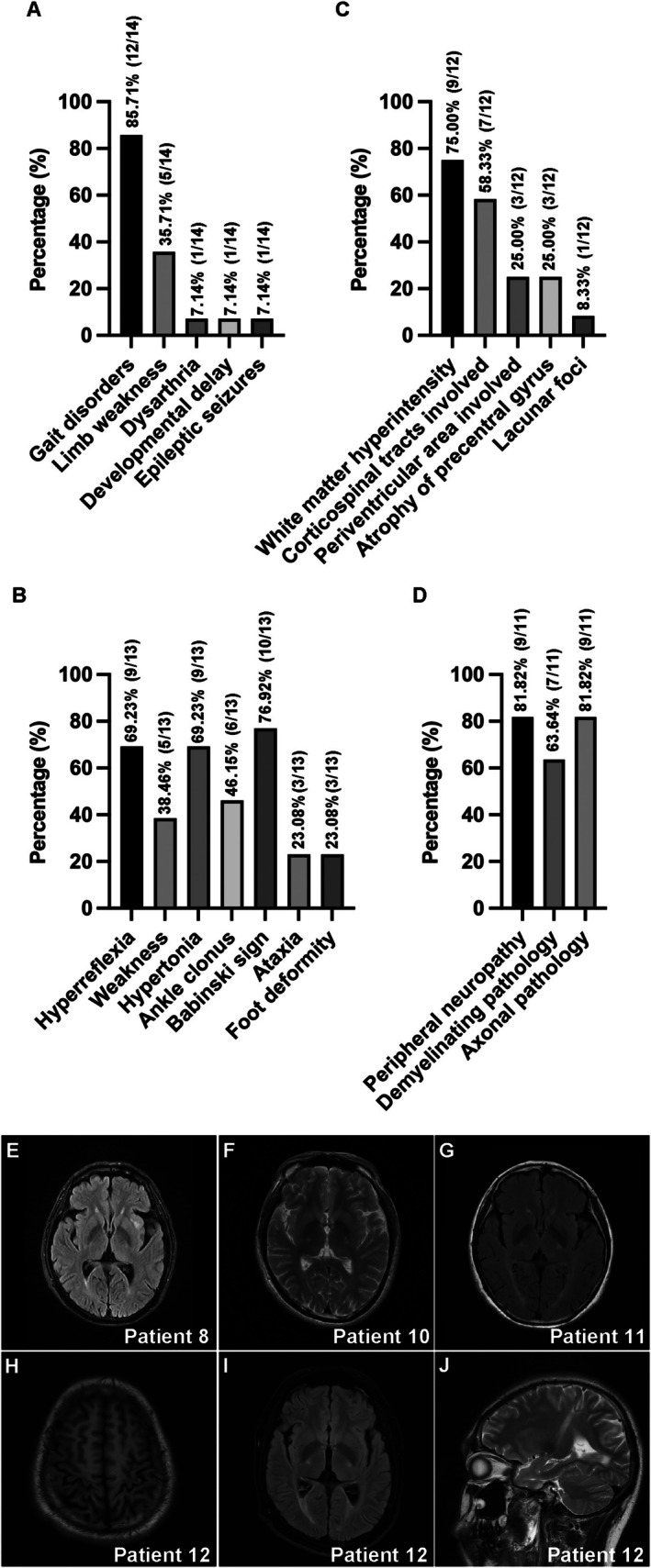
Clinical features and representative MRI of KD patients. (A) Percentage of main clinical manifestations. Gait disorders were the most common symptom in adult KD patients. (B) Percentage of positive physical signs. The most prevalent finding was a positive Babinski sign, followed by hyperreflexia and hypertonia. (C) Percentage of main neuroimaging features. Most patients showed WMH, primarily affecting the corticospinal tracts and periventricular areas. (D) Percentage of main electrodiagnostic features. Most patients showed peripheral neuropathy with demyelinating and axonal pathology. (E–G) T2‐weighted MRI of patients 8, 10, and 11 showing WMH in the corticospinal tracts. Patient 8 also showed WMH in the left insular and subcortical frontal lobes. (H) T1‐weighted MRI of patient 12 showing bilateral precentral gyrus atrophy. (I, J) T2‐weighted MRI of patient 12 showing periventricular WMH (I), and corticospinal tract degeneration (J). KD, Krabbe disease; WMH, white matter hyperintensity.

Proband 2 presented with classic infantile‐onset KD, experiencing epileptic seizures at 12 months of age accompanied by developmental regression and progressive motor decline, particularly affecting gait stability and coordination. The adolescent/adult‐onset group exhibited a more insidious disease course, with symptom onset between 16 and 48 years of age (Figure 5A). Gait disturbance was the most common initial complaint, reported by 11 of 14 individuals (78.57%). These abnormalities were often accompanied by lower limb spasticity, postural instability, and difficulty squatting. A subset of patients (4/14, 28.57%) reported proximal lower limb weakness, reflecting underlying pyramidal tract involvement. Upper limb impairment was relatively uncommon (2/14, 14.28%); however, proband 12 experienced gradual loss of manual dexterity, and proband 6 uniquely presented with isolated upper limb weakness and sensory disturbances, suggesting a distinct pattern of central‐peripheral nervous system involvement.

Among the 13 patients who underwent physical examination (Table S1 and Figure 5B), the most prevalent finding was a positive Babinski sign (10/13, 76.92%), followed by hyperreflexia and hypertonia (9/13, 69.23%). Ankle clonus was present in 46.15% (6/13) of patients, while weakness was detected in 38.46% (5/13). Less common findings included ataxia and foot deformity, each present in 23.08% (3/13) of the examined patients. This constellation of physical signs suggests predominant involvement of the pyramidal tracts in conjunction with peripheral neuropathies.

Notably, proband 6 carrying *GALC* variants of c.136G>T (p.D46Y) and c.1019C>T (p.P340L) was a 56‐year‐old male with a chronic, asymmetrical history of progressive weakness, presenting initially in the right upper limb over 10 years ago, followed by involvement of the left upper limb 5 years later. Physical examination revealed significant motor impairment in the upper extremities, with muscle strength graded 3/5, accompanied by diminished tendon reflexes. Muscle tone remained normal in all four limbs. This clinical picture was strongly supported by electrophysiological studies, which were critical for characterizing the neuropathy. The nerve conduction studies indicated a primary demyelinating pathology predominantly affecting the proximal segments of the nerves in all four limbs. This was compounded by significant secondary axonal degeneration, which was multifocal and notably more severe in the upper limbs. The process also involved sensory nerves, confirming a sensorimotor polyneuropathy. Despite the profound upper limb involvement and widespread neurophysiological abnormalities, lower limb function remains intact with normal gait. This presentation differs from classic KD, where pyramidal tract damage predominates. The asymmetry of peripheral nerve damage is also relatively rare. Considerable heterogeneity in *GALC* mutations may underlie these phenotypic differences.

### 3.6 Neuroimaging and Electrodiagnostic Features of KD Probands

Brain MRI abnormalities were documented in 12 cases to illustrate typical radiological manifestations of KD (Table 3 and Figure 5C), with representative images from probands 8, 10, 11, and 12 presented in Figure 5E–J. The majority of probands (9/12, 75%) exhibited white matter hyperintensity (WMH), most frequently involving the corticospinal tracts (7/12, 58.33%), a radiological hallmark of KD. Additionally, WMH was noted in the bilateral parietal lobes and adjacent to the lateral ventricles in proband 4, and in the left insular region and subcortical frontal lobes in proband 8, indicating variable regional involvement of cerebral white matter. Three probands (3/12, 25.00%) showed signs of atrophy in the precentral gyrus, which correlates anatomically with motor cortex degeneration and clinically with progressive motor deficits. Notably, four probands exhibited normal brain MRI scans despite manifesting clinical symptoms, underscoring the diagnostic challenge of imaging‐negative KD, particularly in late‐onset presentations.

**TABLE 3:** Neuroimaging and electrodiagnostic features of 14 patients with Krabbe disease.

Patient No.	Brain MRI	Electromyography
1^a^	No visible abnormalities	Peripheral neurogenic damage affecting both sensory and motor fibers, with primary demyelination accompanied by axonal damage
2^a^	Diffused white matter lesions	Primarily demyelinating with associated axonal loss of the right common peroneal nerve, accompanied by bilateral superficial peroneal sensory axonal damage
3^a^	WMH of bilateral corticospinal tracts, localized atrophy of the precentral gyrus	Widespread symmetrical motor and sensory peripheral neuropathies in all four limbs, mainly demyelinating lesions with secondary sensory axonopathy
4	Striped high signals in bilateral parietal lobe and lateral ventricles	Neurogenic axonal damage in right lower limb
5^a^	No visible abnormalities	NA
6	NA	Proximal demyelinating neuropathy of all four limbs with multifocal axonal damage in both upper extremities, affecting sensory nerves
7	NA	NA
8	Striped high signals in the corticospinal tracts, slight white matter hyperintensity in the left insular and subcortical frontal lobes	Peripheral neuropathic damage in all four limbs, affecting both sensory and motor fibers, characterized by demyelination with axonal loss
9	No visible abnormalities	No obvious abnormalities
10	Right parafalcine meningioma, WMH of bilateral corticospinal tracts	Demyelinating pathology in the right median nerve, axonal damage in the left superficial peroneal nerve, indicative of multifocal peripheral nerve involvement
11	WMH of bilateral corticospinal tract, mild atrophy of the bilateral precentral gyrus, bilateral frontal lacunar foci	Axonal damage in the motor nerves in lower limbs
12	Bilateral atrophy of the precentral gyrus, corticospinal tract degeneration changes, periventricular WMH	Multifocal peripheral neurogenic damage in all four limbs, primarily demyelinating with sensory axonal loss
13	Striped high signals in the right temporal lobe	No obvious abnormalities
14	WMH of bilateral corticospinal tract	NA

*Note:* Novel variant is in bold.

Abbreviations: NA, not available; WMH, white matter hyperintensity.

^a^ Previously reported patients.

Among the 11 probands who underwent electromyography (Table 3 and Figure 5D), the majority exhibited clear electrophysiological evidence of peripheral neuropathy (9/11, 81.82%). A predominant pattern of mixed demyelinating and axonal pathology was observed, with seven probands (7/11, 63.64%) exhibiting features of primary demyelination accompanied by secondary axonal damage. This was frequently characterized as a widespread, symmetrical sensorimotor polyneuropathy affecting all four limbs. Two patients showed a more focal presentation, involving the right common peroneal nerve or a multifocal pattern. Two patients had electrophysiological studies within normal limits, showing no evidence of neurogenic or myopathic abnormalities. The findings suggest widespread peripheral nerve involvement within this cohort, with a strong propensity for combined demyelinating‐axonal pathology.

## 4 Discussion

Krabbe disease (KD) is a rare lysosomal storage disorder characterized by diverse clinical and genetic manifestations. To date, over 300 variants have been documented in the HGMD [17]. While much of the existing literature focuses on infantile‐onset KD, comprehensive epidemiological data on adult‐onset KD in China remain scarce. Addressing this gap, our study reports 14 unrelated Chinese KD patients and identifies 11 *GALC* variants, including a previously unreported variant in HGMD c.1019C>T (p.P340L), thereby expanding the clinical and genetic spectrum of the disease in this population.

When considered alongside recent multicenter studies from Korean and Japanese cohorts [16, 18, 19, 20], our findings highlight a distinct genotypic profile of KD in East Asian populations compared to those of Caucasian descent. Notably, the 30‐kb deletion, the most common variant among Europeans, followed by p.T529M and p.Y567S [21], is nearly absent in East Asian patients. Instead, missense variants predominate in this region. In Chinese patients, p.P154H and p.L634S are the most frequent variants associated with infantile‐ and adult‐onset KD, respectively [16]. Consistent with reports from Korea and Japan, p.L634S represents a mutational hotspot in our cohort as well.

Previous genotype–phenotype analyses have largely centered on the correlation between variant type and age at onset or disease severity. Truncating mutations, such as large deletions, frameshift, or nonsense variants, typically lead to severe phenotypes, whereas missense variants often associate with later onset, milder symptoms, and slower progression [22]. This trend is exemplified by p.L634S, whose clinical impact is modulated by the variant on the trans allele. Combinations with another missense variant usually result in later‐onset disease, whereas pairing with a truncating variant often leads to earlier and more severe manifestations. Our observations align with this model and are supported by functional studies indicating that missense variants may preserve residual enzyme activity or disrupt trafficking without complete functional loss, thereby attenuating disease progression. Nevertheless, more detailed genotype–phenotype correlations warrant further investigation. Continued identification and functional characterization of novel *GALC* variants will enhance our understanding of disease mechanisms and facilitate personalized therapeutic strategies.

The diagnosis of KD, particularly the adult‐onset form, remains challenging due to symptomatic overlap with other neurological disorders, such as hereditary spastic paraplegia (HSP), Kennedy disease, and spinocerebellar ataxia (SCA). HSP is a group of genetic disorders characterized by progressive spasticity and weakness, primarily in the lower limbs. MRI of HSP patients may also reveal white matter changes [23]. Similarly, KD frequently presents with gait disturbance and spasticity. In our center, most adult‐onset KD cases were initially misdiagnosed as HSP. Kennedy disease, an X‐linked disorder caused by an androgen receptor gene mutation, leads to progressive bulbar and limb muscle weakness [24]. Although motor symptoms resemble those of KD, Kennedy disease typically spares central white matter and often normal MRI and is associated with elevated creatine kinase [24]. These features help distinguish the two conditions. Both Krabbe disease and SCA can present with ataxic gait, muscle weakness, and coordination difficulties. In particular, cerebellar ataxia may be seen in both conditions, leading to confusion in early diagnosis [25]. However, KD is typically more progressive and is accompanied by cognitive decline and characteristic white matter changes on MRI, which are uncommon in most ataxias. Krabbe disease may also be mistaken for other neurodegenerative disorders that cause dementia, motor dysfunction, and ataxia, such as multiple system atrophy (MSA), Parkinson's disease, or Alzheimer's disease in elderly patients. Although symptoms may overlap, these disorders differ in underlying pathology and disease course. Given these diagnostic challenges, genetic testing for *GALC* variants is essential for a definitive diagnosis. Brain MRI also offers valuable clues, particularly when white matter abnormalities are present. Expanding awareness of these differential diagnoses and ensuring timely diagnostic workups will help improve the diagnosis and management of Krabbe disease, ultimately leading to better patient outcomes.

The pathogenicity of *GALC* mutations involves diverse mechanisms depending on their location and nature. For example, the mutations p.T529M, p.L634S, and p.L645R affect GALC trafficking and alter lysosomal localization and secretion [3, 26, 27]. Missense mutations at the p.T112 and p.R396 residues, which are essential for substrate binding, impair GALC activity instead of trafficking [8, 28, 29]. These findings reveal distinct functional consequences of different mutations. In this study, we confirmed the pathogenic effects of p.D56H and p.P340L. Further functional studies of other variants will be crucial for a complete understanding of their roles in disease.

Currently, there is no cure for KD. Management relies on supportive care to alleviate symptoms and maintain quality of life. However, recent therapeutic advances offer renewed hope. Hematopoietic stem cell transplantation (HSCT) is the only established treatment for KD and has shown benefit in infants treated before the onset of significant neurological impairment [30]. By supplying a source of functional GALC enzyme, HSCT can slow disease progression in some cases. Its efficacy in adult‐onset KD, however, remains uncertain, largely due to irreversible nervous system damage at later stages. Other emerging strategies, including gene therapy and enzyme replacement therapy, also hold promise [31, 32]. Preclinical studies of AAV‐mediated gene therapy have demonstrated increased GALC expression and extended survival in KD models. Clinical trials evaluating AAV‐based therapies (e.g., NCT04693598, NCT05739643) and HSCT in infantile KD are currently underway [33]. However, safety concerns regarding systemic AAV delivery remain a barrier to clinical application. Continued research into disease mechanisms and advances in therapy development may lead to more effective interventions, and possibly a cure in the future.

## 5 Conclusion

In summary, this study presents a comprehensive analysis of the genetic and clinical spectrum of Krabbe disease in a Chinese cohort, with a particular focus on adult‐onset cases. We identified 11 *GALC* variants, including the novel p.P340L mutation, and provided functional evidence supporting its pathogenicity through impaired protein processing, mislocalization, and reduced enzymatic activity. The clinical heterogeneity observed highlights the diagnostic challenge of adult‐onset KD. Our findings expand the mutational and phenotypic landscape of KD in the Chinese population, improve understanding of genotype–phenotype correlations, and underscore the importance of functional studies in variant interpretation. This work facilitates earlier diagnosis, informed genetic counseling, and lays groundwork for future targeted therapies.

## Funding

This work was supported by grant (No. 82230062) from the National Natural Science Foundation to Zhi‐Ying Wu, and the Research Foundation for Distinguished Scholar of Zhejiang University to Zhi‐Ying Wu (188020‐193810101/089).

## Ethics Statement

This study was approved by the Ethics Committee of the Second Affiliated Hospital, Zhejiang University School of Medicine (No. 2015‐048).

## Consent

Each participant signed a written informed consent.

## Conflicts of Interest

The authors declare no conflicts of interest.

## Supporting information


**Table S1:** Physical examinations of 14 patients with Krabbe disease.

## Data Availability

The data that support the findings of this study are available from the corresponding author upon reasonable request.
