# Supplementary material for: Genetic and Clinical Characteristics of Chinese Adult Patients With Krabbe Disease
Source: CNS Neurosci Ther. 2025 Dec 18;31(12):e70708. doi: 10.1002/cns.70708 (PMC12715268; doi:10.1002/cns.70708)
Supplement: Supplementary file 1 — Table S1: Physical examinations of 14 patients with Krabbe disease. [file CNS-31-e70708-s001.docx]

**Supplementary Table 1 Physical examinations of 14 patients with Krabbe disease**

| **Patient No.** | **Gender** | Deep tendon reflex (LUL/RUL/LLL/RLL) | Muscle strength (LUL/RUL/LLL/RLL) | Muscle tone (LUL/RUL/LLL/RLL) | Ankle clonus (L/R) | Babinski sign (L/R) | Ataxia | Foot deformity |
| --- | --- | --- | --- | --- | --- | --- | --- | --- |
| 1† | Male | +++/+++/+++/+++ | 5/5/5/5 | -/- | +/+ | +/- | - | + |
| 2† | Male | ++++/+++/++++/+++ | 5/5/5/5 | -/↑ | +/- | +/- | + | - |
| 3† | Female | ++/++/++/++ | 5/5/4/4 | -/- | -/- | +/+ | + | + |
| 4 | Male | ++/++/++/++ | 5/5/4/4 | -/↑ | -/- | -/- | - | - |
| 5† | Male | ++++/++++/++++/++++ | 5/5/4/4 | -/↑ | +/+ | +/+ | - | - |
| 6 | Male | +/+/++/++ | 3/2/5/5 | -/- | -/- | -/- | - | - |
| 7 | Male | NA |  |  |  |  |  |  |
| 8 | Male | ++/++/+++/+++ | 5/5/5/5 | ↑/↑ | -/- | +/+ | - | - |
| 9 | Female | +++/+++/+++/+++ | 5/5/5/5 | ↑/↑ | +/+ | +/+ | - | + |
| 10 | Male | ++++/++++/++++/++++ | 5/5/5/5 | ↑/↑ | +/+ | +/+ | - | - |
| 11 | Female | +++/+++/++++/+++ | 5/5/4/5 | ↑/↑ | -/- | +/+ | + | - |
| 12 | Male | ++++/++++/++++/++++ | 5/5/5/5 | ↑/↑ | +/+ | +/+ | - | - |
| 13 | Female | ++/++/++/++ | 5/5/5/5 | -/- | -/- | -/- | - | - |
| 14 | Female | ++++/++++/++++/++++ | 5/5/5/5 | -/↑ | -/- | +/+ | - | - |

Previously reported patients are marked with †. LUL, left upper limb; RUL, right upper limb; LLL, left lower limb; RLL, right lower limb; L, left; R, right; NA, not available.
